# A meta-analysis of influencing factors of non-suicidal self-injury behavior in adolescents with depression

**DOI:** 10.3389/fpsyt.2026.1758127

**Published:** 2026-07-13

**Authors:** Yanfei Zheng, Xudong Zhao, Leiwen Tang

**Affiliations:** 1 Department of Nursing, The Second Affiliated Hospital Zhejiang University School of Medicine, Hangzhou, Zhejiang, China; 2 Huzhou Third Municipal Hospital, Huzhou, Zhejiang, China

**Keywords:** adolescent depression, influencing factors, meta-analysis, non-suicidal self-injury (NSSI) behavior, nursing

## Abstract

**Aim:**

This study aimed to analyze the influencing factors of non-suicidal self-injury (NSSI) in adolescents with depression.

**Methods:**

This systematic review and meta-analysis were conducted in accordance with PRISMA guidelines. We systematically searched PubMed, Embase, Web of Science, the Cochrane Library, and the China National Knowledge Infrastructure (CNKI) for observational studies on factors influencing NSSI behavior in adolescents with depression published between 1 January 2010 and 7 May 2026. Two reviewers independently screened studies, extracted data, and assessed methodological quality using the Newcastle–Ottawa Scale. Meta-analyses were performed in RevMan 5.4 using mean differences (MDs) with 95% confidence intervals. Between-study heterogeneity was evaluated using the χ² test and I² statistic, and fixed- or random-effects models were applied as appropriate.

**Results:**

In total, 11 studies were included, involving 529 patients in the study group and 1,716 patients in the control group. The meta-analysis showed that, compared with the control group, the study group had significantly higher scores for interpersonal problems, negative emotions, alexithymia, and negative self-esteem [MD = 5.88, 95% CI (3.19, 8.57), P < 0.001], somatic symptom scores [MD = 5.20, 95% CI (–0.03, 10.43), P = 0.05], emotional deficit [MD = 6.82, 95% CI (0.32, 13.31), P = 0.04], Hamilton Depression Rating Scale scores [MD = 2.97, 95% CI (1.76, 4.18), P < 0.001], emotional abuse [MD = 2.10, 95% CI (1.06, 3.13), P < 0.001], physical abuse [MD = 0.64, 95% CI (0.08, 1.20), P = 0.025], sexual abuse [MD = 0.27, 95% CI (0.12, 0.42), P < 0.001], emotional neglect [MD = 1.67, 95% CI (0.37, 2.97), P = 0.009], physical neglect [MD = 0.81, 95% CI (0.43, 1.19), P < 0.001], and Childhood Trauma Questionnaire total scores [MD = 8.93, 95% CI (3.63, 14.24), P < 0.001].

**Conclusion:**

Adolescents with depressive disorders and NSSI tend to exhibit more severe depressive symptoms, maladaptive emotional regulation patterns, negative coping styles, and higher levels of childhood trauma exposure than those without NSSI. These psychosocial and emotional factors may play important roles in the occurrence and maintenance of NSSI behaviors. Early psychological assessment and targeted intervention may help reduce the risk of NSSI in adolescents with depressive disorders.

## Introduction

Non-suicidal self-injury (NSSI) refers to intentional self-inflicted injury to one’s own body tissue without suicidal intent. This kind of behavior is repetitive and deliberate and occurs frequently during adolescence (1, 2). Large-scale systematic reviews have shown that NSSI is highly prevalent in community and school samples of adolescents, with global lifetime prevalence estimates of approximately 20%–22% and 12-month prevalence approaching 20% (3, 4). These meta-analyses also indicate that NSSI typically begins in early to mid-adolescence and is more common among girls than boys (5, 6). In the majority of cases, adolescents with NSSI do not exhibit suicidal intent, and the behavior is generally considered a maladaptive coping strategy for regulating overwhelming negative emotions and psychological distress (7).

Depression is a mental disorder characterized by persistent low mood and loss of interest, and adolescence is a peak period for the onset of depressive disorders (8). Studies have reported that the incidence of NSSI in depressed adolescents is approximately 50% (9), and recent meta-analytic evidence confirms that more than half of adolescents with depression report self-injurious behaviors during their lifetime or within the previous year, with such behaviors markedly increasing the subsequent risk of suicidal ideation and suicide attempts (8, 10). The factors influencing NSSI in adolescents with depression have received considerable attention, and a growing body of evidence has emerged. For example, Zhang et al. reported that alexithymia, loneliness, and resilience were linked to NSSI in adolescents with depression (11). Chen et al. found that borderline personality features, together with emotional regulation capacity, were associated with NSSI in adolescents with depression (12). At the same time, a recent meta-analysis in general adolescent populations identified multiple risk factors for NSSI, including mental disorders, low health literacy, adverse childhood experiences, bullying, problem behaviors, female sex, and physical symptoms (13). For adolescents with depressive disorders specifically, a recent scoping review mapped a wide range of individual, family, and psychosocial correlates of NSSI; however, it mainly provided a narrative synthesis and drew on studies from limited regions (14). Therefore, although many influencing factors have been reported, the existing evidence remains relatively fragmented, and there is still a lack of a comprehensive quantitative meta-analysis focusing on risk and protective factors for NSSI in adolescents with depression. To address this gap, the present study systematically analyzes and summarizes the influencing factors of NSSI in adolescents with depression by pooling available evidence.

## Data and methods

### Study design and reporting

**Figure 1:**
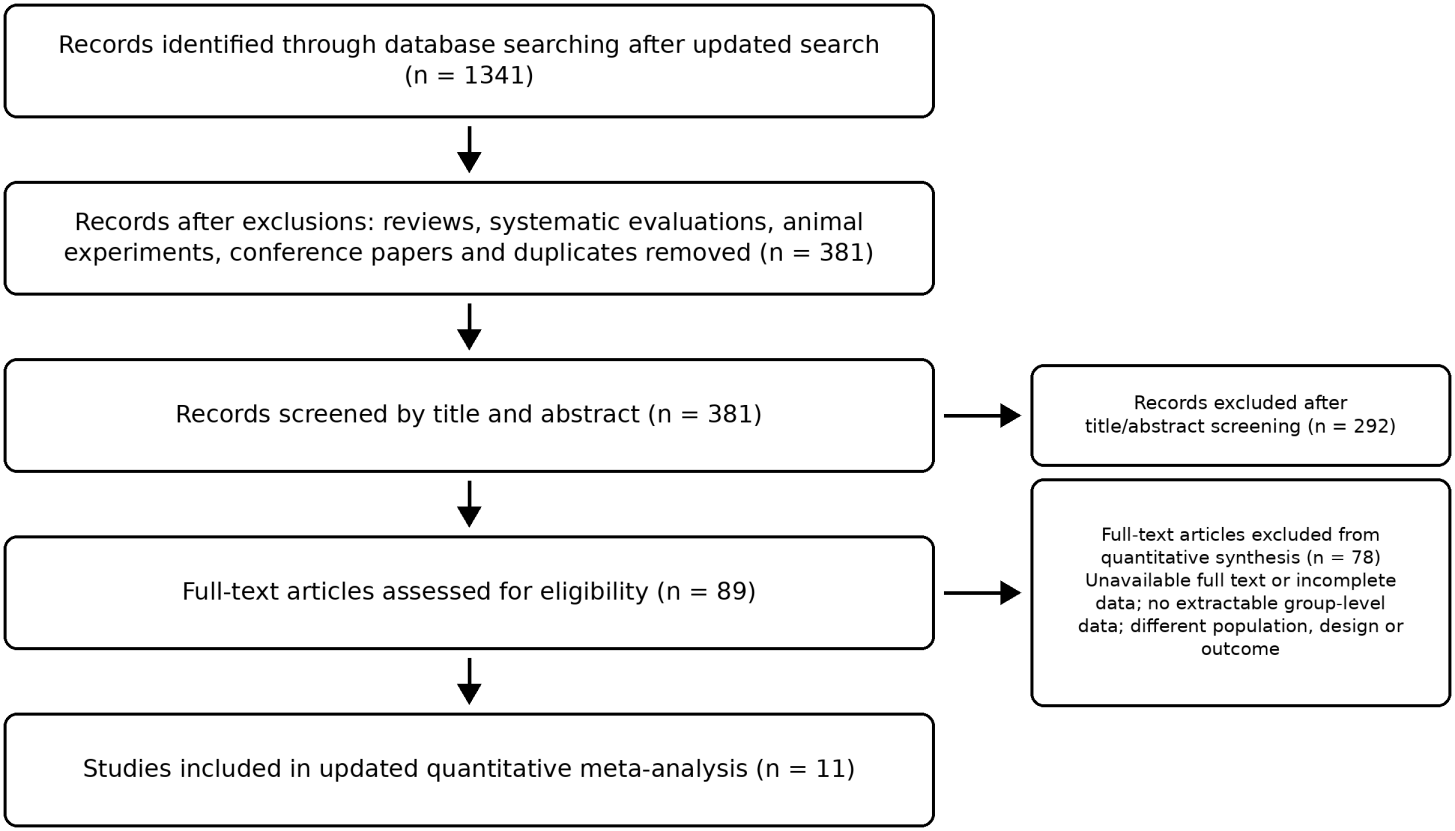
PRISMA 2020 flow diagram of the literature screening and study selection process.

This work is a systematic review and meta-analysis of observational studies. The review was conducted and reported in accordance with the Preferred Reporting Items for Systematic Reviews and Meta-Analyses (PRISMA 2020) statement. A PRISMA 2020 flow diagram summarizes the process of study identification, screening, eligibility assessment, and inclusion (**Figure 1**).

### Inclusion and exclusion criteria

This meta-analysis was framed using the PICO(S) approach. The Population (P) comprised adolescents diagnosed with depressive disorders; the Intervention/Exposure (I) was the presence of NSSI behavior; the Comparison (C) comprised adolescents with depression without NSSI; the Outcomes (O) were candidate influencing factors of NSSI (such as interpersonal problems, negative emotions, alexithymia, trauma, and family-related indicators) assessed with validated instruments; and the Study Design (S) consisted of observational studies.

Inclusion criteria (1): Observational studies, including case–control and cross-sectional studies, in which the study population consisted of adolescents with a clinical diagnosis of depression or depressive disorder (2); studies that compared a group of adolescents with depression and NSSI (study group) with a group of adolescents with depression without NSSI (control group) (3); articles reporting quantitative data on at least one putative influencing factor of NSSI (e.g., psychometric scale scores or clearly defined clinical/psychosocial variables) that could be extracted as mean ± standard deviation or as frequencies for meta-analysis; and (4) full-text articles published in peer-reviewed journals in Chinese or English between 1 January 2010 and 7 May 2026.

Exclusion criteria (1): Conference abstracts, review articles, case reports, dissertations, or research reports without primary quantitative data (2); studies in which the primary population consisted of adolescents with other major psychiatric disorders (e.g., schizophrenia, bipolar disorder, autism spectrum disorder, or substance use disorders) rather than depressive disorders (3); studies that did not clearly distinguish NSSI from suicidal self-injury or did not provide separate data for adolescents with depression with and without NSSI; and (4) studies with incomplete abstracts or full-text information that did not allow extraction of the required data.

### Literature search strategy

We conducted a comprehensive literature search of the Cochrane Library, PubMed, Embase, Web of Science, and the China National Knowledge Infrastructure (CNKI) from 1 January 2010 to 7 May 2026. The search strategy was developed according to the PICO(S) framework described above and in accordance with PRISMA recommendations. For each database, we combined controlled vocabulary (e.g., MeSH terms in PubMed) and free-text terms for the Population, Condition, NSSI behavior, and influencing factors. For example, the PubMed search used the following combination: (“Adolescent”[MeSH] OR adolescent* OR teen* OR youth*) AND (“Depressive Disorder”[MeSH] OR depress* OR “major depressive disorder”) AND (“Self-Injurious Behavior”[MeSH] OR “non-suicidal self-injur*” OR “non-suicidal self-harm” OR “NSSI”) AND (“Risk Factors”[MeSH] OR “risk factor*” OR correlate* OR predictor*). Similar keyword combinations, using database-specific subject headings where available, were adapted for Embase, Web of Science and the Cochrane Library. The CNKI search used equivalent Chinese keywords for adolescents, depression, NSSI, and risk factors. Within each concept block, synonyms were combined using the Boolean operator “OR”, whereas the different concept blocks were combined using “AND”. The operator “NOT” was not used in order to avoid inadvertently excluding potentially relevant studies. In addition, we manually screened the reference lists of all included studies and relevant reviews to identify any further eligible articles.

### Literature screening and data extraction

Two researchers (Y.F.Z. and X. D. Z.) independently screened the titles and abstracts of all records identified by the search, followed by full-text assessment of potentially eligible articles. All references were managed using EndNote, and duplicate records were removed before screening. Disagreements between the two reviewers at any stage were resolved through discussion; when necessary, a third reviewer (L.W.T.) was consulted, and full consensus was reached for all included studies. Because the number of included studies was small and no unresolved discrepancies remained after consensus discussion, formal kappa statistics for inter-observer agreement were not calculated; however, agreement was ensured through the independent screening and consensus process. For each included study, two reviewers independently extracted data using a predefined form, including the first author, year of publication, country or region, study design, diagnostic criteria for depression, definition of NSSI, sample sizes of the NSSI group and non-NSSI control group, participant characteristics (such as age and sex), measurement tools for candidate influencing factors, and quantitative data required for meta-analysis (means and standard deviations for continuous variables or event counts and sample sizes for categorical variables). Any inconsistencies in the extracted data were resolved by re-checking the original articles and reaching a consensus.

All included studies were observational studies that compared adolescents with depression and NSSI with adolescents with depression without NSSI; no randomized controlled trials or quasi-experimental intervention studies were identified or included. Reasons for full-text exclusion (such as wrong population, wrong study design, lack of separate data for NSSI and non-NSSI groups, or incomplete outcome data) were recorded, and the number of excluded records is summarized in the PRISMA 2020 flow diagram (**Figure 1**). The extracted continuous outcome data mainly included mean scores and standard deviations derived from validated psychological assessment instruments, including 24-item Hamilton Depression Rating Scale (HAMD-24), Childhood Trauma Questionnaire (CTQ), Early Adolescent Temperament Questionnaire-Psychosomatic Symptoms Subscale (EATQ-PSS), Early Adolescent Temperament Questionnaire-Affective Subscale (EATQ-AS), and other standardized scales assessing emotional regulation, alexithymia, interpersonal problems, and coping styles.

### Outcome measures and assessment tools

To facilitate interpretation of the pooled effect sizes, the principal validated instruments used across the included studies are briefly described below, including their score ranges and, where applicable, established diagnostic cutoffs.

#### Hamilton Depression Rating Scale

The 24-item Hamilton Depression Rating Scale is a clinician-administered instrument for evaluating depressive symptom severity. Total scores range from 0 to 76. Established severity thresholds are as follows: <8, no clinically significant depression; 8–19, mild depression; 20–34, moderate depression; and ≥35, severe depression. Higher scores indicate greater symptom severity.

#### Childhood trauma questionnaire – short form

The CTQ-SF is a 28-item retrospective self-report measure (25 trauma items plus 3 minimization/denial validity items) assessing five subtypes of childhood maltreatment: emotional abuse, physical abuse, sexual abuse, emotional neglect, and physical neglect. Each subscale comprises five items rated on a 5-point Likert scale (1 = never to 5 = very often), yielding subscale scores of 5–25 and a total score of 25–125. Recommended cutoffs for moderate-to-severe maltreatment are: emotional abuse ≥13, physical abuse ≥10, sexual abuse ≥8, emotional neglect ≥15, and physical neglect ≥10. Higher scores indicate greater maltreatment exposure.

#### Scales for interpersonal problems, negative emotions, alexithymia, and negative self-esteem

Across the included studies, interpersonal problems were assessed using the Inventory of Interpersonal Problems (IIP) or equivalent validated instruments; alexithymia was measured using the Toronto Alexithymia Scale (TAS-20, score range 20–100; cutoff ≥61 indicating alexithymia); negative self-esteem was assessed using the Rosenberg Self-Esteem Scale (RSES, score range 10–40 when reverse-scored for negative self-esteem; lower scores indicate lower self-esteem); and negative emotions were assessed using validated subscales from depressive symptom inventories. In all cases, higher composite or subscale scores indicate greater severity of the respective construct.

#### Somatic symptoms (EATQ-PSS) and emotional deficits (EATQ-AS)

Somatic symptoms and emotional deficits were derived from validated adolescent temperament and affect questionnaires used across the included Chinese clinical studies (denoted EATQ-PSS for the Psychosomatic Symptoms Subscale and EATQ-AS for the Affective Subscale). Higher subscale scores indicate greater severity of somatic complaints or emotional deficits, respectively. The specific score ranges and normative cutoffs for these subscales are reported in the original studies.

#### Negative coping scale

Negative coping strategies were assessed using the Coping Style Questionnaire (CSQ) or equivalent validated instruments. The negative coping subscale typically contains multiple items rated on Likert-type scales; higher subscale scores indicate greater reliance on maladaptive or negative coping strategies.

### Quality assessment and risk of bias

The methodological quality and risk of bias of the included observational studies were evaluated using the Newcastle–Ottawa Scale (NOS) (15). Two reviewers independently rated each study on selection, comparability, and outcome (or exposure) domains, and any discrepancies were resolved through discussion. Studies with a total NOS score of ≥ 6 points were regarded as being of high quality.

### Statistical analysis

Statistical analyses were performed using RevMan 5.4 software. Mean differences (MDs) with 95% confidence intervals (CIs) were used as the effect size measure for continuous variables when identical or equivalent assessment scales were applied across studies. Because most included studies used consistent measurement instruments for the same influencing factors, including the Hamilton Depression Rating Scale (HAMD-24), CTQ, EATQ-PSS, EATQ-AS, and validated psychological scales assessing alexithymia, interpersonal problems, emotional deficits, and negative coping, pooled analyses were conducted using MD as the effect size measure. Heterogeneity among studies was evaluated using the I² statistic and Q test. A fixed-effects model was applied when I² < 50% and P > 0.10; otherwise, a random-effects model was used. Statistical significance was defined as P < 0.05. It should be noted that a statistically significant MD indicates that the NSSI group scored significantly higher than the non-NSSI group on a given scale, but does not necessarily imply that either group’s mean falls within or outside a specific clinical threshold. Both groups may present with elevated scores on the same scale, with the NSSI group scoring significantly higher. Accordingly, the pooled MDs in this meta-analysis should be interpreted as measures of between-group differences rather than as direct indicators of the absolute clinical burden or the presence or absence of a risk factor. Where established diagnostic cutoffs exist for the included scales (e.g., CTQ-SF subscale cutoffs for moderate-to-severe maltreatment and HAMD-24 severity thresholds), readers are encouraged to consider both the absolute mean values reported in each original study and the pooled MD when interpreting clinical significance. Future studies reporting frequencies at validated cutoffs would allow the calculation of odds ratios and further characterize the clinical magnitude of these associations.

## Results

### Literature screening results

A total of 1,341 records were identified through database searching. After the removal of duplicates and non-original articles, followed by title/abstract screening and full-text eligibility assessment, 11 studies met the inclusion criteria for meta-analysis (**Figure 1**). There were 529 patients in the study group (SG) and 1,716 patients in the control group (CG).

### Basic characteristics and quality evaluation of the included studies

The main characteristics and key influencing-factor domains of the 11 included case-control studies, namely, the first author, year of publication, country/region, study design, sample sizes of the NSSI and non-NSSI groups, and the specific psychosocial and clinical domains analyzed, are summarized in **Table 1**. The results of the methodological quality assessment using the Newcastle–Ottawa Scale are presented in **Table 2**. All studies scored ≥ 7 points, indicating generally high quality.

**Table 1:** Basic characteristics and main influencing-factor domains of the included studies.

First author (year)	Country/region	Study design	NSSI group (n)	Non-NSSI group (n)	Main influencing-factor domains analyzed*
Julia A. C. Case (2021)	United States	Case–control study	36	85	Interpersonal problems, negative emotions, alexithymia, and negative self-esteem; Somatic symptoms (EATQ-PSS); Emotional deficits; Negative coping (EATQ-AS)
Yujiao Wen (2021)	China	Case–control study	18	21	Depression severity (HAMD-24); Childhood emotional abuse (CTQ); Childhood physical abuse (CTQ); Childhood sexual abuse (CTQ); Childhood emotional neglect (CTQ); Childhood physical neglect (CTQ); Childhood Trauma Questionnaire (CTQ) total score
Bo Peng (2023)	China	Case–control study	64	60	Interpersonal problems, negative emotions, alexithymia, and negative self-esteem; Somatic symptoms (EATQ-PSS); Emotional deficits; Childhood Trauma Questionnaire (CTQ) total score
Yi Zhou (2022)	China	Case–control study	25	25	Interpersonal problems, negative emotions, alexithymia, and negative self-esteem; Somatic symptoms (EATQ-PSS); Emotional deficits; Negative coping (EATQ-AS)
Huishan Liu (2022)	China	Case–control study	62	44	Somatic symptoms (EATQ-PSS); Emotional deficits; Depression severity (HAMD-24)
Maria Serra (2022)	Italy	Case–control study	31	25	Interpersonal problems, negative emotions, alexithymia, and negative self-esteem; Childhood Trauma Questionnaire (CTQ) total score
Bo Peng (2022)	China	Case–control study	46	80	Interpersonal problems, negative emotions, alexithymia, and negative self-esteem; Childhood emotional abuse (CTQ); Childhood physical abuse (CTQ); Childhood sexual abuse (CTQ); Childhood emotional neglect (CTQ); Childhood physical neglect (CTQ); Childhood Trauma Questionnaire (CTQ) total score
So Yung Yang (2022)	South Korea	Case–control study	104	87	Somatic symptoms (EATQ-PSS); Childhood Trauma Questionnaire (CTQ) total score
Holly C. Wilcox (2012)	United States	Case–control study	75	1006	Childhood physical abuse (CTQ); Childhood sexual abuse (CTQ)
Panpan Cao (2024)	China	Case–control study	127	98	Depression severity (HAMD-24); Childhood emotional abuse (CTQ); Childhood physical abuse (CTQ); Childhood sexual abuse (CTQ); Childhood emotional neglect (CTQ); Childhood physical neglect (CTQ); Childhood Trauma Questionnaire (CTQ) total score
Tian Ren (2025)	China	Case–control study	103	84	Depression severity (HAMD-24); Childhood emotional abuse (CTQ); Childhood physical abuse (CTQ); Childhood sexual abuse (CTQ); Childhood emotional neglect (CTQ); Childhood physical neglect (CTQ); Childhood Trauma Questionnaire (CTQ) total score

Interpersonal problems, negative emotions, alexithymia, and negative self-esteem were assessed using the relevant subscales of depressive symptom questionnaires; somatic symptom scores were derived from EATQ-PSS or equivalent somatic symptom scales; emotional deficits and negative coping were assessed using validated psychological scales; depression severity was measured using the Hamilton Depression Rating Scale (HAMD-24); childhood abuse, neglect, and total trauma exposure were measured using the Childhood Trauma Questionnaire (CTQ) or equivalent instruments.

**Table 2:** Methodological quality of the included studies assessed by the Newcastle–Ottawa Scale.

First author (year)	Study design	NOS score (0–9)
Julia A. C. Case (2021)	Case–control study	7
Yujiao Wen (2021)	Case–control study	8
Bo Peng (2023)	Case–control study	9
Yi Zhou (2022)	Case–control study	9
Huishan Liu (2022)	Case–control study	8
Maria Serra (2022)	Case–control study	8
Bo Peng (2022)	Case–control study	8
So Yung Yang (2022)	Case–control study	9
Holly C. Wilcox (2012)	Case–control study	7
Panpan Cao (2024)	Case–control study	8
Tian Ren (2025)	Case–control study	8

### Meta-analysis results

#### Interpersonal problems, negative emotions, alexithymia and negative self-esteem

**Figure 2:**
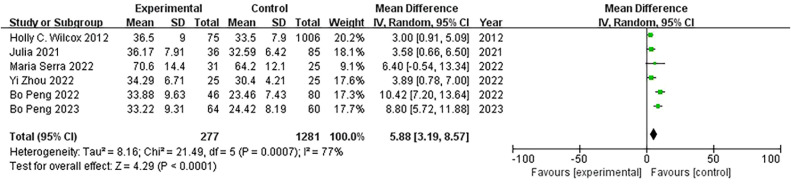
Forest plot of interpersonal problems, negative emotions, alexithymia, and negative self-esteem in the two groups.

A total of six studies were included (16–21), comprising 277 patients in the SG and 1,281 patients in the CG. Heterogeneity test results showed that I^2^ = 77%, P = 0.0007, indicating that the results of the two groups of studies had certain heterogeneity. Therefore, a random-effects model was used for meta-analysis. Compared with the CG, the SG had higher scores for interpersonal problems, negative emotions, alexithymia, and negative self-esteem [MD = 5.88, 95% CI (3.19, 8.57)], showing a statistically significant difference (P < 0.001; **Figure 2**).

### Somatic symptoms (EATQ-PSS)

**Figure 3:**
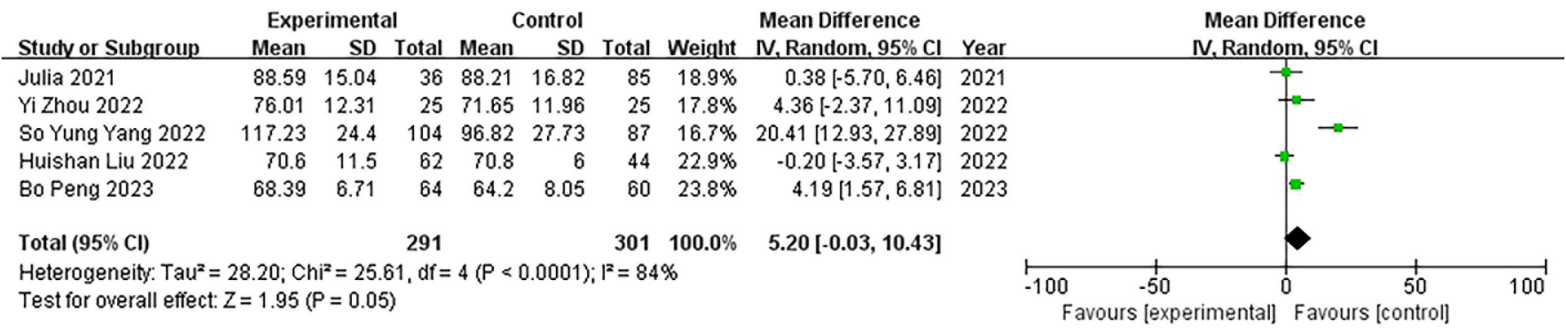
Forest plot of somatic symptom (EATQ-PSS) scores in the two groups.

A total of five studies were included (16–18, 22, 23), involving 291 patients in the SG and 301 patients in the CG. The heterogeneity test showed that I² = 84%, P < 0.001, indicating substantial heterogeneity among the included studies. Therefore, a random-effects model was used for meta-analysis. Compared with the CG, the SG had significantly higher somatic symptom scores [MD = 5.20, 95% CI (–0.03, 10.43), P = 0.05] (**Figure 3**).

### Emotional deficits

**Figure 4:**
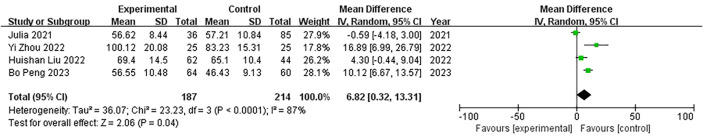
Forest plot of emotional deficit scores in the two groups.

A total of four studies were included (16–18, 22), comprising 187 patients in the SG and 214 patients in the CG. Heterogeneity test results showed that I^2^ = 87%, P < 0.001, indicating that the results of the two groups had certain heterogeneity. Therefore, a random-effects model was used for meta-analysis. Compared with the CG, the SG had a higher score for emotional deficits [MD = 6.82, 95% CI (0.32, 13.31)], showing a statistically significant difference (P = 0.04, **Figure 4**).

### Negative coping

**Figure 5:**
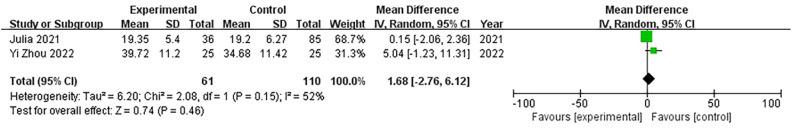
Forest plot of negative coping scores in the two groups.

Two studies were included (16, 18), comprising 61 patients in the SG and 110 patients in the CG. Heterogeneity test results showed that I^2^ = 52%, P = 0.15, reflecting that the results of the two groups had certain heterogeneity. Therefore, a random-effects model was used for meta-analysis. Compared with the CG, the SG had a higher negative coping score [MD = 1.68, 95% CI (–2.76, 6.12)], but the difference was not statistically significant (P = 0.46) (**Figure 5**).

### Hamilton Depression

**Figure 6:**
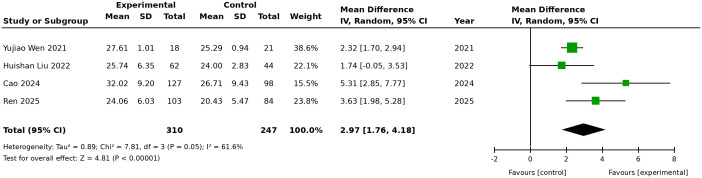
Forest plot of Hamilton Depression scores in the two groups.

A total of four studies were included (22, 24–26), comprising 310 patients in the SG and 247 patients in the CG. The heterogeneity test showed that I² = 61.6%, P = 0.050, indicating moderate heterogeneity among the included studies. Therefore, a random-effects model was used for meta-analysis. Compared with the CG, the SG showed significantly higher Hamilton depression scores [MD = 2.97, 95% CI (1.76, 4.18), P < 0.001] (**Figure 6**).

### Emotional abuse

**Figure 7:**
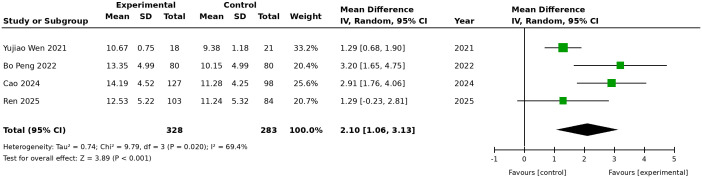
Forest plot of emotional abuse scores in the two groups.

A total of four studies were included (17, 24–26), comprising 328 patients in the SG and 283 patients in the CG. The heterogeneity test showed that I² = 69.4%, P = 0.039, indicating moderate heterogeneity among the included studies. Therefore, a random-effects model was used for meta-analysis. Compared with the CG, the SG had significantly higher emotional abuse scores [MD = 2.10, 95% CI (1.06, 3.13), P < 0.001] (**Figure 7**).

### Physical abuse

**Figure 8:**
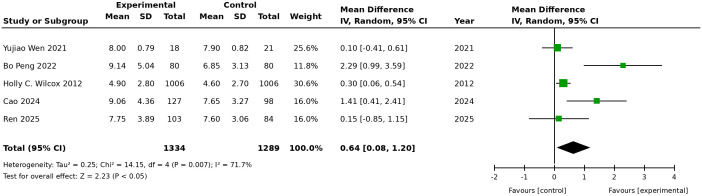
Forest plot of physical abuse scores in the two groups.

A total of five studies were included (17, 21, 24–26), comprising 1,334 patients in the SG and 1,289 patients in the CG. The heterogeneity test showed that I² = 71.7%, P = 0.014, indicating substantial heterogeneity among the included studies. Therefore, a random-effects model was used for meta-analysis. Compared with the CG, the SG had significantly higher physical abuse scores [MD = 0.64, 95% CI (0.08, 1.20), P = 0.025] (**Figure 8**).

### Sexual abuse

**Figure 9:**
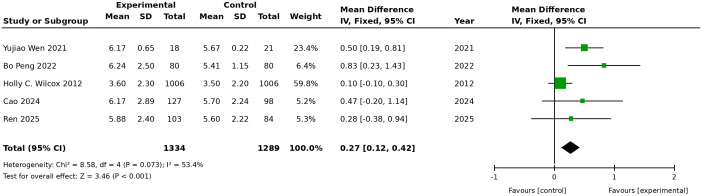
Forest plot of sexual abuse scores in the two groups.

A total of five studies were included (17, 21, 24–26), comprising 1,334 patients in the SG and 1,289 patients in the CG. The heterogeneity test showed that I² = 53.4%, P = 0.072. According to the predefined analysis strategy, a fixed-effects model was used for meta-analysis. Compared with the CG, the SG had significantly higher sexual abuse scores [MD = 0.27, 95% CI (0.12, 0.42), P < 0.001] (**Figure 9**).

### Emotional neglect

**Figure 10:**
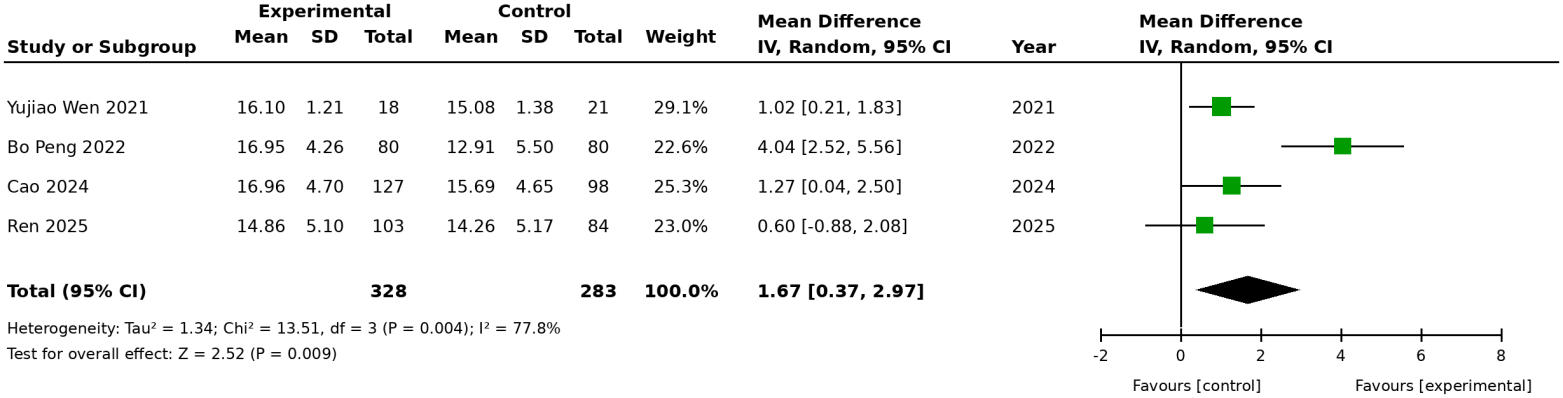
Forest plot of emotional neglect scores in the two groups.

A total of four papers (17, 24–26) were included, comprising 328 patients in the SG and 283 patients in the CG. The heterogeneity test showed that I² = 77.8%, P = 0.004, indicating substantial heterogeneity among the included studies. Therefore, a random-effects model was used for meta-analysis. Compared with the CG, the SG had significantly higher emotional neglect scores [MD = 1.67, 95% CI (0.37, 2.97), P = 0.009] (**Figure 10**).

### Physical neglect

**Figure 11:**
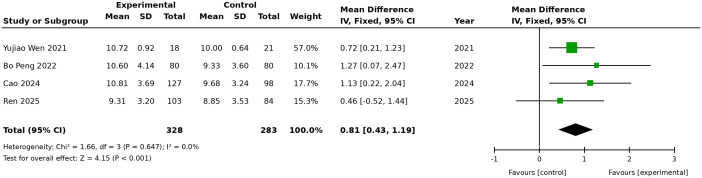
Forest plot of physical neglect scores in the two groups.

A total of four papers were included (17, 24–26), comprising 328 patients in the SG and 283 patients in the CG. The heterogeneity test showed that I² = 0.0%, P = 0.774, indicating no significant heterogeneity among the included studies. Therefore, a fixed-effects model was used for meta-analysis. Compared with the CG, the SG had significantly higher physical neglect scores [MD = 0.81, 95% CI (0.43, 1.19), P < 0.001] (**Figure 11**).

### Overall childhood maltreatment

**Figure 12:**
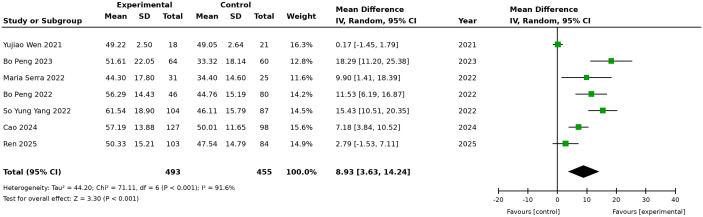
Forest plot of Childhood Trauma Questionnaire (CTQ) total scores in the two groups.

Seven studies (17, 19, 20, 23–26) were included, comprising 529 patients in the SG and 455 patients in the CG. The heterogeneity test showed that I² = 91.6%, P < 0.001, indicating substantial heterogeneity among the included studies. Therefore, a random-effects model was used for meta-analysis. Compared with the CG, the SG had significantly higher CTQ total scores [MD = 8.93, 95% CI (3.63, 14.24), P < 0.001] (**Figure 12**).

## Discussion

There are cultural and ethnic differences in the occurrence of NSSI behavior, and relevant data indicate that its prevalence is higher in developing countries than in developed countries. The reported prevalence of NSSI ranges from 12% to 45% among American adolescents, 10% to 22% among Canadian college students, and 11% to 23% among Chinese adolescents (27). Research reports have shown that the reported prevalence of NSSI among adolescents is gradually increasing, indicating that NSSI has become an important problem influencing adolescents’ behavioral health and warrants greater attention, especially in the group of adolescents with depression, where the reported prevalence of NSSI is higher (28, 29). Therefore, it is particularly important to analyze the factors associated with NSSI in adolescents with depression. NSSI behavior is one of the strongest factors in predicting future suicidal behavior. However, domestic research or parents’ understanding of this behavior is still not comprehensive enough, and it is believed that the occurrence of this behavior is only one of the ways that adolescents use to attempt to meet their emotional needs, and its treatment may sometimes be delayed, which may have a serious impact on the healthy development of adolescents. Therefore, effective intervention for adolescents with depression to reduce the risk of NSSI is conducive to promoting the healthy development of adolescents.

### Overview of main findings

The present meta-analysis demonstrated that adolescents with depressive disorders and NSSI showed significantly higher Hamilton Depression scores, emotional abuse scores, physical abuse scores, sexual abuse scores, emotional neglect scores, physical neglect scores, and CTQ total scores than adolescents without NSSI. In addition, these patients also exhibited more severe interpersonal problems, negative emotions, alexithymia, and negative coping tendencies. These findings suggest that childhood trauma exposure and maladaptive emotional processing may be closely associated with the development of NSSI behaviors in adolescents with depressive disorders. Our findings are generally consistent with the previous meta-analysis conducted by Niu et al. (30), which also suggested that depressive severity, emotional dysregulation, and childhood trauma may be important factors associated with NSSI in adolescents with depressive disorders. The present study further updates and refines the available evidence regarding these influencing factors.

### Interpersonal and emotional factors

First, our analyses showed that adolescents with depression and NSSI had significantly higher scores for interpersonal problems, negative emotions, alexithymia, and negative self-esteem compared with adolescents with depression without NSSI. From a theoretical perspective, NSSI has been widely conceptualized as a maladaptive strategy for regulating intense negative affect and coping with interpersonal distress (31, 32). Difficulties in identifying, expressing, and regulating emotions, combined with low self-worth, may lead adolescents to engage in NSSI to reduce emotional tension, punish themselves, or seek attention and support from others (33). Our results are supported by previous empirical studies and meta-analyses (13, 34). Klonsky’s functional model of NSSI highlights emotion regulation, self-punishment, and interpersonal functions as central motives for self-injury (35). A recent meta-analysis identified emotional dysregulation, poor coping, and low self-esteem as important risk factors for NSSI among adolescents in general (36). In addition, a systematic review in adults found a robust negative association between self-esteem and NSSI, suggesting that low self-esteem is a transdiagnostic correlate of self-injurious behaviors (37, 38). For adolescents with depressive disorders specifically, our findings suggest that emotional and interpersonal vulnerabilities are not only correlates of depression but may also distinguish those who go on to engage in NSSI (39, 40). Clinically, these findings support the use of comprehensive psychosocial assessments that include interpersonal functioning, emotion regulation skills, and self-esteem rather than focusing solely on depressive symptoms (36, 41). Targeted interventions such as cognitive-behavioral therapy to modify negative self-beliefs, dialectical behavior therapy skills training to enhance emotion regulation, and family-based or school-based programs to improve communication and support may therefore be particularly important in preventing and reducing NSSI among adolescents with depression (41, 42).

### Somatic symptoms, emotional deficits, and depressive severity

Second, we observed that adolescents with depression and NSSI had higher scores on somatic symptom scales and emotional deficit measures, along with higher Hamilton Depression scores, than adolescents with depression without NSSI. These findings may be rational because somatic complaints and emotional blunting may reflect more severe or atypical depressive psychopathology, including heightened physiological arousal, alexithymia, and difficulties in experiencing positive emotions, which in turn may increase the likelihood that adolescents resort to NSSI as a way to counteract emotional numbness or to regulate distressing internal states (35, 43). Several previous studies support these results (36, 39). Neurobiological and neurophysiological research in adolescents with depression and NSSI has reported alterations in prefrontal cortex activity, reward processing, and stress-response systems, which may contribute to both somatic symptom amplification and emotional numbing (44, 45). Clinical studies have also shown that higher depressive symptom severity is associated with an increased risk of NSSI and suicidal behavior in young people (46, 47). Our findings support the interpretation that somatic symptoms, emotional deficits, and higher overall depressive severity are not merely by-products of depression but may be markers of a more complex and high-risk clinical profile in adolescents who self-injure. Clinicians should therefore pay close attention to atypical or prominent somatic complaints, emotional blunting, and high depression scores when assessing adolescents with depression, and consider integrating interventions that target both mood symptoms and associated somatic and neurocognitive dysfunction (e.g., psychoeducation on somatic manifestations of depression, relaxation and stress-management training, and structured behavioral activation) (42, 43).

### Childhood abuse and neglect

Third, our meta-analyses found that adolescents with depression and NSSI had significantly higher levels of emotional abuse, sexual abuse, physical neglect, and combined indices of somatic abuse, emotional abuse, emotional neglect, and somatic neglect than adolescents with depression without NSSI. Emotional abuse, physical abuse, sexual abuse, emotional neglect, and physical neglect were all significantly associated with NSSI behaviors. Early abuse and neglect can disrupt attachment, emotion regulation, and self-concept, leading to maladaptive coping strategies such as NSSI in the face of stress (48, 49). Our findings are in line with previous research showing robust associations between childhood maltreatment and NSSI (50). Martin et al. reported that childhood abuse and neglect were linked to non-suicidal self-injury via insecure attachment and maladaptive internal working models (48). A recent meta-analysis also identified adverse childhood experiences, including various forms of abuse and neglect, as key risk factors for NSSI among adolescents (51). Moreover, studies in adolescents with depression have highlighted that childhood trauma is common and may contribute to more severe clinical presentations, including self-injurious behavior (48, 52). Based on this convergence of evidence, we interpret childhood maltreatment not only as a distal risk factor but also as a contextual factor that interacts with current depressive symptoms, interpersonal difficulties, and emotion-regulation problems to increase the likelihood of NSSI.

### Clinical and community implications

Beyond statistical significance, the present findings have several important implications for clinical practice and community-based prevention. The risk factors identified in this meta-analysis—such as interpersonal problems, emotional dysregulation, alexithymia, negative self-esteem, and histories of abuse or neglect—can be incorporated into routine risk screening in child and adolescent mental health services, school psychological counseling, and community health settings. Multidisciplinary teams should develop structured assessment protocols that integrate these domains, rather than relying solely on symptom checklists for depression. In terms of interventions, our results support the use of targeted psychological approaches that combine emotion regulation training, cognitive restructuring of negative self-beliefs, enhancement of interpersonal skills, and trauma-informed interventions that address the impact of childhood maltreatment. At the community level, school-based mental health promotion, anti-bullying programs, parenting support, and public education about NSSI may help to reduce stigma, facilitate early identification of at-risk adolescents, and promote timely referral for treatment.

### Study limitations

Although this meta-analysis synthesized the available evidence regarding factors associated with NSSI in adolescents with depression, several limitations should be considered when interpreting the findings. First, only 11 case–control studies with relatively small sample sizes were included, which may reduce the statistical power and affect the stability of some pooled estimates. Second, all included studies were observational studies, predominantly single-center case–control designs; therefore, causal relationships between the identified influencing factors and NSSI could not be established. Third, considerable heterogeneity was observed in several pooled analyses. Potential sources of heterogeneity include differences in psychometric assessment tools, diagnostic criteria for depression, operational definitions of NSSI, sample characteristics, cultural and regional backgrounds, and methodological variations across studies. In addition, some psychological constructs, such as emotional deficits and variables related to emotional regulation, were assessed using different validated scales across studies, which may also have contributed to between-study variability. Fourth, because most included studies used consistent assessment instruments, pooled analyses were primarily conducted using mean differences rather than standardized mean differences, which improved the interpretability of the pooled effect estimates; however, residual methodological heterogeneity could not be completely excluded. Fifth, most included studies were conducted in East Asian populations, particularly in Chinese clinical settings, which may limit the generalizability of the findings to other ethnic, cultural, and healthcare contexts. Finally, publication bias could not be completely ruled out, as gray literature and unpublished negative studies may not have been fully captured despite the comprehensive search strategy.

### Future research directions

Future research should build on these results by conducting large-scale, multicenter prospective cohort studies to clarify the temporal relationships between risk factors and NSSI and to explore potential mediating and moderating mechanisms. Community-based randomized or quasi-experimental intervention trials are also needed to evaluate the effectiveness of multicomponent prevention programs that integrate school, family, and healthcare resources for high-risk adolescents with depressive disorders. In addition, cross-cultural comparative studies would help determine whether the pattern of influencing factors identified in this meta-analysis is applicable to adolescents with depression in different countries and cultural backgrounds and to tailor intervention strategies accordingly.

## Conclusion

Interpersonal problems, negative emotions, alexithymia, negative self-esteem, somatic symptoms, depressive severity, childhood abuse, neglect, and overall childhood trauma are important influencing factors of NSSI in adolescents with depression. In clinical settings, close attention should be paid to the emotional changes in adolescents with depression, and timely psychological intervention should be implemented.

## Data Availability

The original contributions presented in the study are included in the article/supplementary material. Further inquiries can be directed to the corresponding author.
